# Correlation between tumor marker CA72-4 and prognosis of patients with gastric cancer: A protocol for systematic review and meta-analysis

**DOI:** 10.1097/MD.0000000000023723

**Published:** 2020-12-24

**Authors:** Minxia Li, Fei Xue, Jie Yang, Xiaodong Pan

**Affiliations:** a The People's Hospital of Danyang city, Danyang, Jiangsu Province; b Dongchang Fu People's Hospital, Liaocheng, Shandong Province; c Central Laboratory, Danyang People's Hospital of Jiangsu Province, Danyang, Jiangsu Province; d Zhangye People's Hospital Affiliated to Hexi University, Zhangye, Gansu Province, China.

**Keywords:** carbohydrate antigen 72-4, gastric cancer, prognosis, protocol, systematic review

## Abstract

**Background:**

Gastric cancer is one of the common gastrointestinal tumors, with high recurrence and metastasis rates. Tumor marker tumor marker carbohydrate antigen 72-4 (CA72-4) has been used in the screening and diagnosis of gastric cancer, but whether it can be used as an indicator to monitor the prognosis of gastric cancer remains a great controversy. The purpose of this study was to systematically evaluate the correlation between tumor marker CA72-4 and prognosis of gastric cancer patients.

**Methods:**

A systematic search was performed by retrieving on English databases (PubMed, Embase, Web of Science, the Cochrane Library) and Chinese databases (China Knowledge Network, Wanfang, Weipu (VIP Information Chinese Journal Service Platform), CBM) of clinical study on the correlation between tumor marker CA72-4 and prognosis of gastric cancer patients. The retrieval time limit was from the establishment of the database to October 2020. Two researchers independently extracted and evaluated the quality of the data in the included study. A meta-analysis was performed using Stata12.0 and RevMan5.3 software.

**Conclusions:**

This study will compare the correlation between tumor marker CA72-4 and prognosis of gastric cancer patients, so as to provide evidence-based basis for clinicians to select prognostic indicators of gastric cancer.

**Ethics and dissemination:**

Private information from individuals will not be published. This systematic review also does not involve endangering participant rights. Ethical approval was not required. The results may be published in a peer-reviewed journal or disseminated at relevant conferences.

**OSF Registration number:**

DOI: 10.17605 / OSF.IO / B3AMN

## 1 Introduction

Gastric cancer is one of the most common malignant tumors in the world, ranking the fifth in the global incidence rate (5.7%) and the third in the mortality rate (8.2%).^[1]^ Although the development of gastric cancer diagnosis and treatment technology in recent years has prolonged the overall survival (OS) of patients to some extent, most patients were diagnosed with advanced stage at the first diagnosis and often died due to rapid multi-organ metastasis, with a 5-year survival rate of only 5%.^[2]^ Although the level of surgery and chemoradiotherapy for gastric cancer has been constantly improved, the recurrence, metastasis, and mortality rate are still high.^[3,4]^ Therefore, it is of great significance to find sensitive and specific biomarkers to accurately determine the prognosis of gastric cancer patients.

Tumor markers are a kind of substances that can reflect the existence of tumors.^[5,6]^ In recent years, great progress has been made in the study of serum tumor markers related to gastric cancer, but most of the studies of tumor markers are limited to the diagnostic significance of tumors. Whether it can be used as an index to monitor the prognosis of gastric cancer is still controversial and no agreement has been reached so far. Tumor marker carbohydrate antigen 72-4 (CA72-4) is a high molecular glycoprotein antigen, which does not exist in benign tumor tissues, body fluids, and normal tissues. But it can be expressed at a high level in gastrointestinal tumors, pancreatic cancer, endometrial cancer, and ovarian cancer, and has different degrees of specificity for these tumors, especially for gastric cancer. So it is often used as an index for the detection of digestive system tumors.^[7,8]^ Some studies have indicated that serum CA72-4 can be used as a useful marker for the efficacy and prognosis of chemotherapy for gastric cancer,^[9]^ while others have indicated that serum CA72-4 is not a reliable prognostic indicator for gastric cancer.^[10]^

At present, there have been a number of clinical studies on the correlation between tumor marker CA72-4 and prognosis of gastric cancer,^[11–13]^ but whether tumor marker CA72-4 can be used as a prognostic factor of gastric cancer to guide the individualized treatment of gastric cancer patients is still controversial. This systematic evaluation aims to evaluate the correlation between tumor marker CA72-4 and prognosis of gastric cancer based on existing evidence, so as to provide an evidence-based basis for clinicians.

## 2 Methods

### 2.1 Protocol register

This protocol of systematic review and meta-analysis has been drafted under the guidance of the preferred reporting items for systematic reviews and meta-analyses protocols.^[14]^ Moreover, it has been registered on open science framework on November 13, 2020 (Registration number: DOI: 10.17605 / OSF.IO / B3AMN).

### 2.2 Ethics

Since this is a protocol with no patient recruitment and personal information collection, the approval of the ethics committee is not required.

### 2.3 Inclusion criteria

(1) Patients who were clearly diagnosed as gastric cancer by pathological examination, and treated with operation, chemoradiotherapy, and other regimens;
(2) A cohort study which studies the correlation between tumor marker CA72-4 and prognosis of gastric cancer;
(3) The CA72-4 test specimens derived from peripheral blood;
(4) Follow-up data which includes OS, progression-free survival or disease-free survival;
(5) Language limited to Chinese and English

### 2.4 Exclusion criteria

(1) Studies published repeatedly;
(2) Studies whose literature are abstract or data are incomplete, or whose data could not be obtained after contacting the author;
(3) Conference summaries, comments, abstracts, reviews, case reports, animal experiments, etc.

### 2.5 Search strategy

“CA72-4”(CA72-4), “Gastric cancer”(wei ai), “Stomach neoplasm”(wei zhong liu) were used for retrieval in Chinese databases, including China Knowledge Network, Wanfang Data Knowledge Service Platform, VIP Information Chinese Journal Service Platform, and China Biomedical Database. English retrieval words such as “CA72-4”, “CA724”, “Stomach cancer”, “Gastric cancer” were used for retrieval in English databases, including PubMed, EMBASE, Web of Science and the Cochrane Library. The retrieval time was from the establishment of the database to October 2020, and all the domestic and foreign literatures about studies on the correlation between tumor marker CA72-4 and prognosis of gastric cancer patients were collected. Take PubMed as an example, and the retrieval strategy is shown in Table 1.

**Table 1:** Search strategy in PubMed database.

Number	Search terms
#1	carbohydrate antigen 72-4 [title/abstract]
#2	CA72-4 [title/abstract]
#3	CA724 [title/abstract]
#4	#1 or #2 or #3
#5	Stomach neoplasm [MeSH]
#6	Neoplasm, stomach [title/abstract]
#7	Gastric neoplasm [title/abstract]
#8	Neoplasm, gastric [title/abstract]
#9	Cancer of stomach [title/abstract]
#10	Gastric cancer [title/abstract]
#11	Cancer, gastric [title/abstract]
#12	Stomach cancer [title/abstract]
#13	Cancer, stomach [title/abstract]
#14	#5 or #6 or #7 or #8 or #9 or #10 or #11 or #12 or #13
#15	#4 and #14

### 2.6 Data screening and extraction

**Figure 1:**
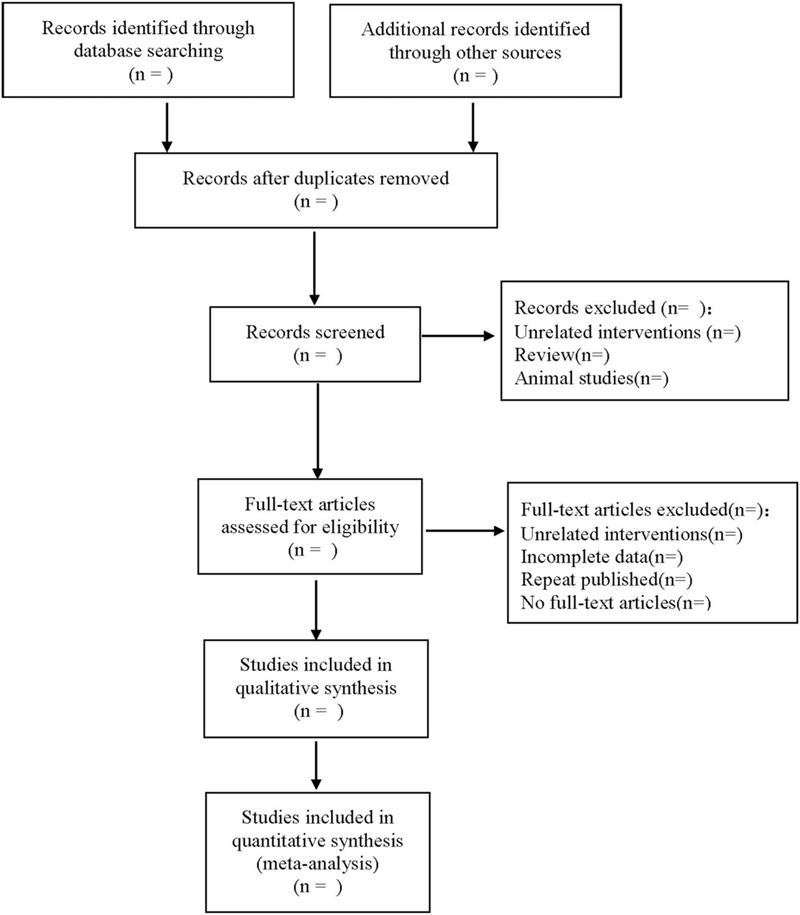
Flow diagram.

The 2 researchers independently extracted the following data by reading the literature: first author, country and year of publication, sex and age of the included patients, number of cases, follow-up time, tumor stage, cut-off value of CA72-4, OS, progression-free survival or HR, and 95%CI of disease-free survival. The data were extracted and cross-checked by 2 researchers independently. Disagreements are resolved through discussion or with the assistance of a third investigator. The literature screening process is shown in Figure 1.

### 2.7 Literature quality assessment

According to the Newcastle-Ottawa Scale, the quality evaluation of the included research was carried out,^[15]^ including 3 columns and 8 items with a total score of 9:

(1) Selection of study population: appropriateness of case determination (1 point); representativeness of case (1 point); selection of control group (1 point); determination of control group (1 point);
(2) Comparability between 2 groups: comparability between case and control group, which was considered in design and statistical analysis (1 point);
(3) Measurement of exposure factors: determination of exposure factors (1 point); same method used to determine exposure factors between the case and the control group (1 point); no response rate (1 point).

Scores were given by the 2 researchers term by term according to the performance of the included literature in the above evaluation items, and cross-checked after completion respectively. In case of any disagreement, discussion was required. If no agreement could be reached, a decision would be made in consultation with researchers from the third party.

### 2.8 Statistical analysis

#### 2.8.1 Data analysis and processing

This meta-analysis was performed using RevMan 5.3 software. HR and the corresponding 95% CI or P value were used to evaluate the prognosis, with OS as the primary outcome. Heterogeneity in included studies was assessed using Cochran's Q test and Higgins I^2^, if *P*≥.1, I^2^≤50%, there was low inter-study heterogeneity, and the fixed-effect model was adopted. If *P*<.1, I^2^>50%, it indicated inter-study heterogeneity and should explore the source of heterogeneity and the random-effects model was adopted. If more than 10 studies were performed, funnel plots were used to evaluate the existence of publication bias. Moreover, Egger and Begg test were used for the evaluation of potential publication bias.

#### 2.8.2 Dealing with missing data

If the data of the required study is incomplete or not reported in the study, the researcher will contact the first author or other author by phone or email. If the required data are not available, we will use descriptive analysis instead of meta-analysis or exclude these studies if necessary.

#### 2.8.3 Subgroup analysis

In this study, a subgroup analysis will be conducted according to the cut-off value of CA72-4. Due to the heterogeneity that may result from ethnic differences, a subgroup analysis will be conducted according to the source of literature.

#### 2.8.4 Sensitivity analysis

As recommended by the Cochrane handbook, sensitivity analysis of each indicator is required. In order to test the stability of meta-analysis results of indicators, a one-by-one elimination method will be adopted for sensitivity analysis.

#### 2.8.5 Grading the quality of evidence

We will use Grading of Recommendation Assessment, Development and Evaluation scoring method to grade the evidence of the outcome index.^[16]^ The evidence will be downgraded by bias risk, indirectness, inconsistency, inaccuracy, publication bias and upgraded by large effect, Plausible confounding, Would change the effect, and Does-response Gradient. The quality of evidence will be rated as high, medium, low, or very low eventually.

## 3 Discussion

Tumor markers are substances produced by the host in response to tumor stimulation or by abnormal secretion of malignant tumor cells, which generally exist in tumor cells in the form of metabolites such as antigens, enzymes, hormones, and so on. In normal tissues or benign lesions, tumor markers are hardly or infrequently produced, but the content in tumor tissues is significantly beyond the normal range. Besides, the more advanced it is, the higher secretion of tumor markers level is.^[17]^ Clinically, relevant tumors can be identified and diagnosed through their biochemical or immune characteristics. Due to its simple operation and non-invasive nature, it is easier to be applied to the general survey, early diagnosis and efficacy monitoring of healthy people and people with high risk of gastric cancer. CA72-4 is a mucin-like high molecular weight glycoprotein which is released during the continuous expansion of tumor cells. Its molecular weight is 220 to 400 kD and the content is < 6 U/mL in normal human serum.^[18]^ CA72-4 can affect the self-expansion ability of tumor cells and evaluate its expansion rate,^[19]^ which is one of the tumor markers commonly used in the screening and diagnosis of gastric cancer. Studies have shown that CA72-4 is correlated with the depth of tumor invasion, lymph node metastasis, peritoneal metastasis, and distant metastasis.^[20]^ In gastric cancer patients with lymph node, peritoneal, and serous involvement, the positive rate of CA72-4 is higher. The results are of great significance to evaluate the prognosis and survival status of patients with gastric cancer and predict recurrence.^[21]^

CA72-4 is highly sensitive to gastric cancer, and the positive rate of serum CA72-4 in gastric cancer is reported to be 36% to 94%. And its specificity is also high, some of which even reach 100%.^[22]^ Clinical studies have found that serum CA72-4 is lower after the resection of gastric cancer than that before operation, and there is a significant difference before and after operation. As a consequence, CA72-4 can be used to detect whether there are residual tumor cells after operation and judge the prognosis of gastric cancer.^[23]^ Although more and more studies have pointed out the importance of CA72-4 in the prognosis of gastric cancer, there is a lack of evidence-based evidence. In this study, we will summarize the latest evidence of the correlation between CA72-4 and the prognosis of gastric cancer, so as to provide a reliable basis for the application of CA72-4 in the prognosis of gastric cancer.

However, this systematic review has some limitations. The tumor stage and metastasis degree were different in the included studies, and the detection methods of CA72-4 were inconsistent, which may have some clinical heterogeneity. In addition, due to the limitation of language ability, we only search English and Chinese literature and may ignore studies or reports in other languages.

## Author contributions

**Data curation:** Minxia Li, Fei Xue.

**Funding acquisition:** Xiaodong Pan.

**Investigation:** Minxia Li, Fei Xue.

**Software:** Jie Yang.

**Supervision:** Xiaodong Pan.

**Writing – original draft:** Minxia Li, Fei Xue.

**Writing – review & editing:** Minxia Li, Xiaodong Pan.
